# Active Hexose-Correlated Compound Shows Direct and Indirect Effects against Chronic Lymphocytic Leukemia

**DOI:** 10.3390/nu15245138

**Published:** 2023-12-18

**Authors:** Giovanna Merchand-Reyes, Ramasamy Santhanam, Maria L. Valencia-Pena, Krishan Kumar, Xiaokui Mo, Tesfaye Belay, Jennifer A. Woyach, Bethany Mundy-Bosse, Susheela Tridandapani, Jonathan P. Butchar

**Affiliations:** 1 Department of Internal Medicine, Division of Hematology, The Ohio State University, Columbus, OH 43210, USA; giovanna.merchandreyes@osumc.edu (G.M.-R.);; 2 Department of Biomedical Informatics, The Ohio State University, Columbus, OH 43210, USA; 3 Pelotonia Institute for Immuno-Oncology, The Ohio State University Comprehensive Cancer Center, The Ohio State University, Columbus, OH 43210, USA; 4 Department of Applied Sciences and Mathematics, Bluefield State University, Bluefield, WV 24701, USA

**Keywords:** natural compounds, immunotherapy, antibody therapy, chronic lymphocytic leukemia, acute myeloid leukemia

## Abstract

Chronic lymphocytic leukemia (CLL) is a disease characterized by the accumulation of mature CD19^+^CD5^+^CD23^+^ B cells in the bloodstream and in lymphoid organs. It usually affects people over 70 years of age, which limits the options for treatments. The disease is typically well-managed, but to date is still incurable. Hence, the need for novel therapeutic strategies remains. Nurse-like cells (NLCs) are major components of the microenvironment for CLL, supporting tumor cell survival, proliferation, and even drug resistance. They are of myeloid lineage, guided toward differentiating into their tumor-supportive role by the CLL cells themselves. As such, they are analogous to tumor-associated macrophages and represent a major therapeutic target. Previously, it was found that a mushroom extract, Active Hexose-Correlated Compound (AHCC), promoted the death of acute myeloid leukemia cells while preserving normal monocytes. Given these findings, it was asked whether AHCC might have a similar effect on the abnormally differentiated myeloid-lineage NLCs in CLL. CLL-patient PBMCs were treated with AHCC, and it was found that AHCC treatment showed a direct toxic effect against isolated CLL cells. In addition, it significantly reduced the number of tumor-supportive NLCs and altered their phenotype. The effects of AHCC were then tested in the Eµ-TCL1 mouse model of CLL and the Mll^PTD/WT^ Flt3^ITD/WT^ model of AML. Results showed that AHCC not only reduced tumor load and increased survival in the CLL and AML models, but it also enhanced antitumor antibody treatment in the CLL model. These results suggest that AHCC has direct and indirect effects against CLL and that it may be of benefit when combined with existing treatments.

## 1. Introduction

Chronic lymphocytic leukemia (CLL) is a disease characterized by the accumulation of mature CD5^+^CD19^+^CD23^+^ cells in the bloodstream [1]. It is the most common type of leukemia in adults in the United States, with an estimated 18,740 new cases in all age ranges, and approximately 4490 deaths in 2023 [2]. It mainly affects the elderly, with a median age of 70 at the time of diagnosis [3].

There are many options for treating CLL patients. Chemotherapeutic agents include bendamustine, cyclophosphamide, and fludarabine [4]. In addition, several small-molecule inhibitors have been used to block B-cell receptor (BCR) signaling, including the Bruton Tyrosine Kinase (BTK) inhibitor ibrutinib and the phosphoinositol-3 kinase δ (PI3Kδ) inhibitor idelalisib [1]. Rituximab, along with other anti-CD20 monoclonal antibodies (α-CD20), is also commonly employed [4]. There are different combinatorial therapeutic approaches as well, which can be adjusted according to the age of the patient and the presence of specific mutations [4,5]. Unfortunately, not all patients respond to the first line of treatment, with some developing Richter’s transformation, a more aggressive form of lymphoma; most of these transformations result in diffuse large B cell lymphoma (DLBCL) [6]. Thus, although there are increasing resources to treat CLL patients, there is still work to be performed to achieve better responses.

Although malignant, isolated CLL cells have been shown to exhibit short survival times in vitro [7]. Extensive research has found that they are surrounded and supported by cells in the microenvironment at proliferation centers in the spleen and lymph nodes, including T cells, stromal cells, and macrophages, among others. The cells that constitute the microenvironment are possibly recruited actively by CLL cells. In turn, microenvironmental cells interact through cell-to-cell contacts and produce soluble factors that promote the survival of CLL cells, as well as contribute to BCR activation [8].

One of these cell types in the microenvironment, nurse-like cells (NLCs) play a major role in supporting the survival and proliferation of CLL cells. NLCs originate from CD14^+^ monocytes and can be derived in vitro by co-culturing monocytes with CLL cells for 14 days [7,9]. Characterized by the expression of CD68, CD163, and stromal cell-derived factor (SDF)-1, among other markers, NLCs have been equated to tumor-associated macrophages (TAMs) and have been seen to be in close contact with CLL cells in lymphoid tissues of patients [7,10,11]. NLCs provide a variety of survival signals to CLL cells to promote their survival, and they are also known to protect against CLL-cell death caused by agents such as ibrutinib and dexamethasone [7,12,13,14,15,16].

Cancer immunotherapies can help stimulate the immune system to respond more effectively against tumors. Such immune modulators include recombinant cytokines such as interferons, synthetic compounds such as Toll-like receptor (TLR) agonists, and natural products. These are also being explored as potential enhancers of antibody therapy [17,18,19,20]. Regarding natural products, since 2007, there have been numerous novel natural products brought to market, including Ixabepilone (Ixempra^®^) for aggressive breast cancer and Vinflunine (Javlor^®^) for bladder cancer [21].

Active Hexose-Correlated Compound (AHCC) is a mushroom extract derived from several species of *Basidiomycetes* mushrooms, including Shiitake (*Lentinus edodes*) and Shimeji (*Lyophyllum shimeji*) [22]. It contains a mixture of amino acids, minerals, polysaccharides, and lipids enriched in α-1,4-linked glucans [23,24,25,26]. AHCC has improved the prognosis and quality of life of patients with liver, lung, head, and neck cancers [27,28,29]. In addition, it is non-toxic, with high doses eliciting only mild adverse events [26,30].

AHCC is an immunostimulatory agent that has effects on monocytes, natural killer (NK) cells, T cells, and natural killer T (NKT) cells [22,23,31,32]. For example, it promotes cytokine production by intestinal epithelial cells and monocytes [22], increases the production of Tumor Necrosis Factor α, (TNFα), Interleukin 2 (IL-2), Interleukin 6 (IL-6), and Interferon-γ (IFNγ) in a cold-stress and infection model [33] and elicits Interleukin 1β (IL-1β) from monocytes that, in turn, drive T-helper 1 (Th1) and T-helper 17 (Th17) responses [34]. In cancer, AHCC has been shown to increase the number of T cells, NK cells, dendritic cells, and monocytes in different cancer types and models [23,35,36,37,38,39,40]. Nevertheless, despite their importance, the effect of AHCC in tumor-associated macrophages is still scarce.

Here, the effects of AHCC were tested within the context of CLL, and the results showed that it directly impacted the survival of primary patient CLL cells. AHCC also reduced the number of NLCs and induced a slight but measurable change in their general phenotype. In a murine model of CLL, AHCC led to a significant increase in survival time and also enhanced antibody treatment. These results suggest that AHCC has both direct and indirect effects against CLL and that it may be an effective adjuvant for immune-based therapies.

## 2. Materials and Methods

### 2.1. Antibodies and Other Reagents

AHCC was purchased from Quality of Life Labs (Purchase, NY, USA), under recommendation from the manufacturer Amino Up Co., Ltd. (Sapporo, Japan). AHCC was de-waxed for in vitro experiments in accordance with manufacturer instructions, then lyophilized [41]. This was then freshly resuspended in phosphate-buffered saline (PBS, Gibco, Grand Island, NY, USA) for each experiment. Mouse anti-mouse CD20 monoclonal antibody was purchased from Invivogen (San Diego, CA, USA, catalog #mcd20-mab10) and resuspended in PBS before use.

### 2.2. Tissue Culture

Fresh blood samples from CLL patients were provided by consenting patients under an Institutional Review Board (IRB)-approved protocol. Samples were de-identified prior to delivery to the laboratory. Characteristics of each donor is shown in Supplementary Table S1. Peripheral blood mononuclear cells (PBMC) were isolated using lymphocyte separation media (Corning; Corning, NY, USA) by centrifugation at 1500 rpm for 30 min. For nurse-like cell (NLC) development, PBMCs were resuspended in Roswell Park Memorial Institute (RPMI) 1640 media (Gibco) supplemented with 10% heat-inactivated fetal bovine serum (FBS; VWR International, Radnor, PA, USA), 2 mM L-glutamine (Invitrogen, Grand Island, NY, USA), and penicillin/streptomycin (56 U/mL for each; Invitrogen) at a density of 10 × 10^6^ cells/mL. Then, PBMCs were plated in type I collagen coated plates (Corning) at 37 °C in an atmosphere of 5% CO_2_ for 14 days to allow development as previously described [7,9,42].

For CLL cells, PBMCs were washed with incomplete media and resuspended in MACS buffer (Miltenyi Biotec, Bergisch Gladbach, Germany) and further incubated with anti-CD19 magnetic beads (Myltenyi Biotec) on ice for 15 min. Cells were washed again and passed through a magnetic column. CD19^+^ cells were then recovered by pressure, counted, resuspended in complete RPMI media at 3 × 10^6^ cells/mL, and placed in culture plates.

### 2.3. Flow Cytometry

PBMCs from CLL patients were cultured as described above. Cultures were left untreated or treated with AHCC at the specified concentrations at the beginning of the culture. Then, NLCs were allowed to develop. After 14 days, non-adherent cells were collected, and NLCs were detached using type IV collagenase (Worthington Biochemical Corporation, Lakewood, NJ, USA) at 0.1–0.2% in Hanks Buffered Saline Solution (Gibco) supplemented with Polymyxin B (Calbiochem, San Diego, CA, USA) at 10 μg/mL, incubating at 37 °C for 20–60 min. After detachment, both non-adherent and adherent cells were placed back together and washed with PBS. Then, cells were resuspended in a solution of whole human IgG at 10 µg/mL in PBS and incubated in ice for 15 min. Cells were then stained with the following antibodies: anti-human CD14 FITC (clone M5E2; BD Biosciences, Franklin Lakes, NJ, USA), anti-human CD19 Brilliant Violet 421 (clone HIB10; Biolegend, Perkin Elmer, Waltham, MA, USA), anti-human CD163 PeCy7 (clone GHI/61; Biolegend), anti-human CD86 Phycoerythrin (clone IT2.2; Biolegend), and Live/Dead Fixable Blue (Invitrogen). For intracellular markers, cells were fixed and permeabilized using the BD Cytofix/Cytoperm kit (BD Biosciences) according to the manufacturer instructions. After permeabilization, anti-human CD68 Alexa Fluor 785 (clone Y1/82A; Biolegend) and anti-human CXCL12/SDF-1 APC (clone #79018; R&D systems, Minneapolis, MN, USA) were added and incubated for 30 min on ice. Finally, cells were washed and resuspended in PBS with bovine serum albumin (BSA) at 0.5% (Fisher Scientific, Waltham, MA, USA). For acquisition, precision counting beads (Biolegend) were added to get absolute counts. Samples were acquired using the LSR-Fortessa (BD Biosciences) at the Flow Cytometry Shared Resource at The Ohio State University and analyzed using FlowJo version 10.7.2 (Ashland, OR, USA). All appropriate isotype controls were used as negative controls. Geometric mean fluorescence intensities were calculated by subtracting the isotype control value. For measurement of CLL-cell death, isolated CLL cells were treated with AHCC at the specified concentrations at the beginning of the culture and at day 4. Cells were recovered every 24 h, washed, and apoptosis assessed by flow cytometry using Annexin V/PI apoptosis kit (BD Biosciences) according to the manufacturer instructions. Briefly, cells were resuspended in Annexin binding buffer and both Annexin V–FITC and Propidium Iodide were added; cells were incubated at room temperature for 15 min in the dark. Then, more Annexin binding buffer was added along with precision counting beads. As positive single control staining, cells were heated at 55 °C for 10 min and then placed on ice before staining.

### 2.4. Testing of AHCC In Vivo

Mouse experiments were performed under a protocol approved by the Institutional Animal Care and Use Committee at The Ohio State University. Female C57Bl/6 mice aged 6 weeks or 12 weeks were purchased from The Jackson Laboratory (Ban Harbor, ME, USA). Mice were housed in the University vivarium and allowed to acclimate for at least one week prior to commencement of experiments. Splenocytes from diseased Eμ-TCL1 mice (10 × 10^6^) were injected into recipient mice via the tail vein. Treatments began 2–3 weeks after engraftment (21 and 15 days for the first and second experiment, respectively). AHCC was removed from capsules, weighed, suspended in a sterile-PBS slurry, and administered via gavage at 600 mg/kg mouse weight using a plastic feeding tube (Instech Laboratories, Inc., Plymouth Meeting, PA, USA) [33,41]. Gavage volume was 200 μL. Anti-CD20 was administered via intraperitoneal injections at 1 mg/kg mouse weight in 100 μL PBS. Vehicle controls were treated with PBS without (survival experiment) or with (antibody experiment) mouse IgG at 1 mg/kg mouse weight. For the survival experiment, treatment was given until removal, and, for the antibody experiment, the treatments were performed for 7 weeks, or a total of 21 doses. Control mice were non-engrafted mice that received no treatments or vehicles. For white-blood-cell counts, 100 μL blood was drawn by tail-vein bleeding and counted using a 1:20 dilution with the Turk Blood diluting fluid (Ricca Chemical Company, Arlington, TX, USA) in a corrected Neubauer chamber. Survival was counted as time before mice met early removal criteria, which included 20% weight loss, paralysis, inability to stand, uncontrolled shivering, or unwillingness to eat or drink. Specific treatment groups and mouse numbers are below.

For the CLL engraftment survival experiment with versus without AHCC, groups were Vehicle (PBS vehicle gavage, *n* = 6), AHCC-treated (600 mg/kg gavage in PBS, *n* = 6), and non-engrafted controls that received no tumor cells or treatments (*n* = 2). For the CLL engraftment antibody plus AHCC experiment, groups were Vehicle (PBS gavage plus normal mouse IgG at 1 mg/kg delivered intraperitoneally, *n* = 6), AHCC gavage (in PBS at 600 mg/kg plus normal mouse IgG, *n* = 7), mouse anti-mouse CD20 antibody (1 mg/kg delivered intraperitoneally plus PBS gavage, *n* = 7), and AHCC plus mouse anti-mouse CD20 antibody (*n* = 7).

For the acute myeloid leukemia model, the MII^PTD/WT^Flt3^ITD/WT^ mouse model described previously [43,44,45] was used. Wild-type C57BL/6/J-LY5.1 mice were treated by irradiation (8.5 cCy, divided into two doses) to eradicate resident white blood cells and then transplanted with 0.75 × 10^6^ splenocytes from Mll^PTD/WT^Flt3^ITD/WT^-diseased mice. After one week, mice were divided into Vehicle (*n* = 11) and AHCC (*n* = 12) treatment groups, receiving PBS gavage or AHCC (in PBS) gavage at 600 mg/kg, respectively. This was continued until removal criteria were met.

### 2.5. Statistical Analysis

Cell numbers/percents and markers were presented as means or dots at each dose group with standard deviations. Experiments in vitro were designed in a paired fashion such that samples from each patient were used across all treatments. As such, each patient served as its own control. Mixed effect models were used to analyze the data, accounting for the samples from each respective patient being used across different treatments, followed by comparison over vehicle control. Holm’s method (Bonferroni stepdown) was used to adjust *p*-values to control type I error. Residual plots of the statistical models were used to confirm normality. Survival data were demonstrated using Kaplan–Meier curves, and the comparisons of overall survival between groups were analyzed using logrank test. Analyses were performed in SAS 9.4 (SAS institute, Cary, NC, USA).

## 3. Results

### 3.1. AHCC Reduces the Viability of CLL Cells

**Figure 1:**
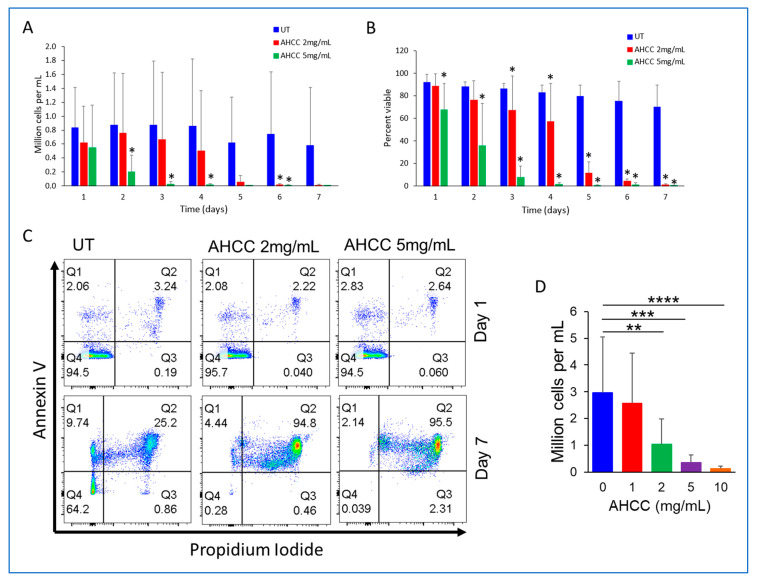
**AHCC directly affects CLL survival.** Isolated CD19^+^ cells from CLL patients were treated at the indicated concentrations of AHCC at the beginning and 4 days after culture. Every day, cells were collected and stained with Annexin V and Propidium Iodide to determine viability. (**A**) absolute counts, as well as (**B**) percent viability, and (**C**) representative dot plots at days 1 and 7 are shown (*n* = 4 donors). (**D**) Patient PBMC (*n* = 7 donors) were isolated and cultured for 14 days following single treatments of 0, 1, 2, 5 or 10 mg/mL AHCC. Cell counts were done at 14 days using flow cytometry. * *p* ≤ 0.05, UT vs AHCC. ** *p* ≤ 0.01, *** *p* ≤ 0.001, **** *p* ≤ 0.0001.

Due to the modulatory and antitumor effects of AHCC, its possible direct effects on CLL-cell viability were first tested. CLL cells were isolated from fresh patient blood samples and treated with AHCC at 2 and 5 mg/mL at the beginning of the culture and again at 96 h. Results showed that AHCC significantly reduced the counts of CLL cells at both concentrations, with 5 mg/mL reducing CLL-cell numbers at day 2 and 2 mg/mL leading to reductions at day 6 (Figure 1A). The percentages of viable cells in each group were also significantly lower. AHCC at 5 mg/mL significantly reduced the percentage of viable CLL cells after 24 h, and 2 mg/mL led to reductions after 3 days (Figure 1B). By Day 7, both concentrations of AHCC led to apoptosis of roughly 95% of CLL cells (Figure 1C). The effects of treating patient PBMC just once with either 0, 1, 2, 5, or 10 mg/mL of AHCC were tested subsequently. On Day 14, cells were examined via flow cytometry. Results showed a concentration-dependent decrease in CLL cell number, with a significant reduction at 2 mg/mL and an almost complete depletion by 10 mg/mL (Figure 1D). Collectively, these data suggest that AHCC directly reduces the survival of CLL cells and that it disrupts viability with even one treatment.

### 3.2. AHCC Reduces NLC Numbers and Alters Their Phenotype

**Figure 2:**
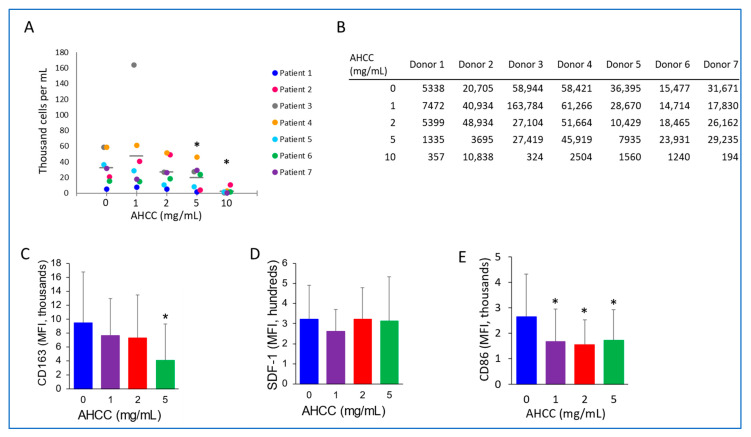
**AHCC reduces NLC numbers and alters their phenotype.** PBMCs isolated from CLL patients were treated with one dose of AHCC at the start of the culture. Cells were incubated for 14 days and the numbers of NLCs (CD14^+^ cells) were measured by flow cytometry using counting beads (graphed in (**A**), with results table in (**B**) Gray bars in (**A**) denote averages. Expression of (**C**) CD163, (**D**) SDF-1 and (**E**) CD86 in CD14^+^ cells was measured using cytometry, with isotype-subtracted mean fluorescence intensity (MFI) shown in graphs (*n* = 7 donors). * *p* ≤ 0.05.

Figure 1D shows the effect of AHCC in a mixed-cell setting, leaving open the possibility that AHCC had indirect effects on CLL cells by affecting other cell types in the culture. It has been shown that AHCC can have immune-stimulating effects on myeloid-lineage cells [22]. Hence, whether this extract had an impact on myeloid-derived NLCs required testing. Using the CLL-patient PBMCs isolated and treated in Figure 1D, the numbers of NLCs, identified as CD14^+^CD68^+^CD163^+^SDF-1^low/+^ cells were quantified. As shown in Figure 2A,B, treatment with 5 and 10 mg/mL AHCC led to a significant reduction. As the numbers of NLCs were greatly reduced at 10 mg/mL, the expressions of different NLC-related markers in CD14^+^CD68^+^ macrophages were evaluated from PBMC cultures treated with AHCC at 1, 2, and 5 mg/mL. Here, results showed that the M2-macrophage-related marker CD163 was significantly reduced at 5 mg/mL, indicating a shift in the phenotype of the NLCs that successfully differentiated with AHCC treatment. NLCs are known for producing stromal cell-derived factor (SDF)-1, a CLL-supportive factor, so we tested that as well but saw no significant change (Figure 2D). AHCC treatment also led to a reduction in the activation marker CD86 (Figure 2E), which has been shown to be expressed in both M1 and M2b macrophages.

### 3.3. AHCC Reduces Leukemic Load While Extending Survival in a Mouse Model of CLL

**Figure 3:**
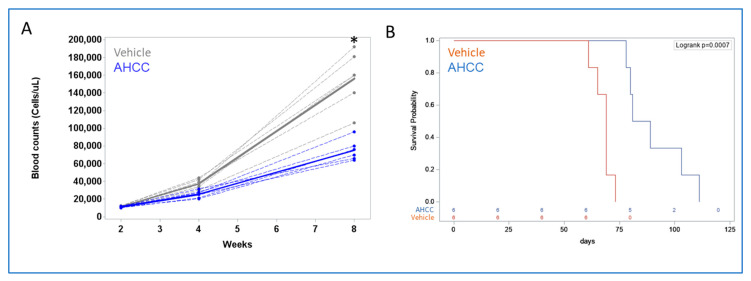
**AHCC reduces leukemic load while extending survival in a mouse model of CLL.** Splenocytes from Eµ-TCL1 mice were injected into 6-week-old healthy female C57Bl/6 mice. After three weeks, mice were treated with vehicle or AHCC at 600 mg/kg by oral gavage three times a week. (**A**) The number of white blood cells was quantified at the specified points. Mean values denoted by thicker solid lines. * The difference between Vehicle and AHCC was significant, *p* < 0.0001. (**B**) Mice were monitored for survival; treatment was continued until removal (*n* = 6 for Vehicle and *n* = 6 for AHCC-treated; *n* = 2 for healthy control). The difference between Vehicle and AHCC was significant, *p* < 0.001.

Next, the effects of AHCC were tested in the Eμ-TCL1 mouse model of CLL, which has been shown to mimic human CLL [46]. Mice were engrafted with splenocytes from diseased mice, then 3 weeks later were treated 3 times per week with either vehicle (PBS) or AHCC via gavage as previously described [33,41]. Counts of white blood cells (WBC) were performed at 2, 4, and 8 weeks. Results showed no differences between treatment groups at weeks 2 and 4, but WBC counts were significantly lower in the AHCC-treated group by week 8 (*p* < 0.0001, Figure 3A). Mice were also followed for survival, and, in concordance with the white-blood-cell counts, AHCC-treated mice showed significantly longer survival (Figure 3B). These results suggest that AHCC can antagonize the progression of CLL in vivo. Similarly, the effect of AHCC on survival in an immune-competent mouse model of acute myeloid leukemia (AML) was tested. As with the CLL model, AHCC treatment significantly extended survival time (Figure S1). These results collectively suggest that AHCC can be beneficial against multiple types of hematologic malignancies.

### 3.4. AHCC Enhances Antibody Effect to Reduce Leukemic Load and Extend Survival In Vivo

**Figure 4:**
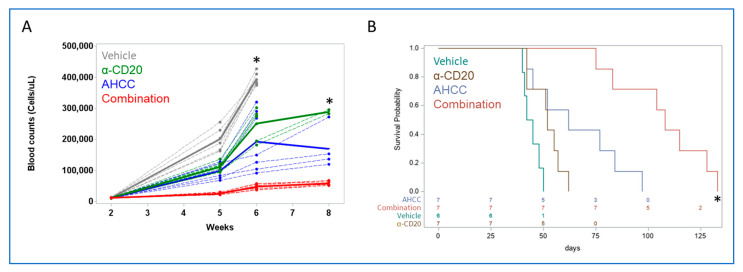
**AHCC enhances antibody effect to reduce leukemic load and extend survival in vivo.** Splenocytes from Eµ-TCL1 mice were injected into healthy 12-week-old female C57Bl/6 mice. After two weeks, mice were treated with vehicle or AHCC at 600 mg/kg by oral gavage, or/and anti-CD20 antibody (α-CD20) at 1 mg/kg intraperitoneally three times a week. Vehicle consisted of PBS gavage and 1 mg/kg normal mouse IgG delivered intraperitoneally. (**A**) The number of white blood cells was quantified at the specified points. Mean values denoted by thicker solid lines. * Differences were significant at week 6 between all treatment groups, and at week 8 between α-CD20, AHCC and Combination, *p* < 0.005. (**B**) After 21 doses, treatment was stopped, and mice followed up for survival (*n* = 6 for Vehicle; *n* = 7 for AHCC; *n* = 7 for α-CD20; *n* = 7 for Combination; *n* = 3 for healthy controls). * All treatments were significantly different from Combination treatment. All control non-engrafted mice survived throughout the experiment.

Due to the abovementioned immune-modulatory properties of AHCC, it was next tested whether it would enhance the effects of immune-based therapy. For this, the mouse model of CLL was again used. Perhaps the most well-known immune-based treatment for CLL is antibody therapy, so it was tested whether AHCC would enhance the antitumor effects of antibody treatment in vivo. Mice were engrafted with diseased splenocytes, then 2 weeks later treated with (1) vehicle, (2) AHCC, (3) mouse anti-mouse CD20 antibody, or (4) AHCC plus antibody. Counts of white blood cells were taken at week 6, then at week 8 with the remaining mice. At week 6, counts were significantly different between all treatment groups, with AHCC-treated mice showing significantly lower counts than vehicle- and antibody-treated groups, and with combination treatment (AHCC plus antibody) leading to significantly lower counts than all other groups (Figure 4A). Treatments were discontinued after week 7 (21 treatments), as most mice in vehicle and antibody-alone groups had met early removal criteria. Although no vehicle-treated mice remained at week 8, comparisons among the other groups showed significant differences between AHCC and antibody, and between each single treatment versus combination treatment (Figure 4A). Similarly, survival was significantly longer in the combination group than in either single-treatment group (Figure 4B). These results suggest that AHCC can significantly enhance the effects of antibody therapy.

## 4. Discussion

Activation of the immune response through natural components that bind to innate receptors has been widely studied and applied to cancer treatment. Particularly, activation of the Toll-like receptors through specific agonists like imiquimod has been used successfully in the clinic [47]. As a natural component, AHCC contains a variety of molecules that have been shown to activate the innate immune response; although the mechanism is not completely understood, it is known that AHCC activates different pattern recognition receptors (PRRs), including TLR4 [22]. 

B cells, specifically CLL cells, are known to express different PRRs, including TLR4 [48]. This suggests that AHCC may have a direct immunomodulatory effect in CLL cells. In the present study, AHCC had a direct, cytotoxic effect on CLL cells, reducing the number of viable cells in vitro, which was observed in cells cultured alone or in co-culture, suggesting an effect in signaling pathways involved in CLL cell survival. It has been shown that Signal Transducer and Activator of Transcription 3 (STAT3) is constitutively phosphorylated in CLL cells and that its de-phosphorylation induces cell death [49,50]; interestingly, AHCC has been shown to reduce STAT3 activation in ovarian cancer, which may be a similar mechanism of action for CLL [51,52]. Another mechanism that may explain the direct effect seen on CLL cells could be activation of apoptosis mediated through Fas, TRAIL, or p38-mediated p53 activation [41,53,54].

In addition to the direct of AHCC on CLL survival, elucidating its effects on one of the major supporters for malignant development, namely, NLCs, was also important. It has previously been shown that AHCC could activate monocytes and monocytic cell lines [22,33,34]. Nevertheless, no report has shown the direct effect of such a compound on tumor-associated macrophages, nor within the context of hematologic malignancies. Although there was a variable response among patient samples, with some showing more sensitivity toward reduction in NLC differentiation after AHCC treatment at lower doses, the trend in AHCC’s effect was significant. In addition, the reduction in NLC numbers with AHCC treatment (Figure 2A) was notable, being significant at both 5 and 10 mg/mL, suggesting that AHCC antagonizes NLC differentiation. Interestingly, an immediate toxic effect in monocytes was not found, which is in line with our previous observation in healthy-donor monocytes [41]. These results suggest that AHCC may interfere more with differentiation than with viability of these myeloid-lineage cells. Whether this is direct or mediated through AHCC’s effect on CLL cells remains to be determined.

Regarding NLC phenotype, the current study also tested whether AHCC could cause a shift toward a less tumor-supportive state. Reduced levels of CD163 were found in macrophages, defined by CD14^+^CD68^+^ cells (Figure 2C). CD163 is a well-known marker for anti-inflammatory macrophages and is usually found in tumor-associated macrophages [55]. In addition, it has been shown that higher expression of CD163 has been related to increased CLL-cell support [56]. On the other hand, levels of SDF-1, previously shown to be a relevant supporter of CLL cells [7], were not substantially affected by AHCC treatment.

Another marker of interest is CD86, which has historically been related to M1-like macrophages. For example, in colorectal cancer, the abundance of macrophages expressing high levels of CD86 and low levels of CD163 could be used as predictor of prognosis [57]. Nevertheless, we found that AHCC significantly downregulated the expression of both CD163 and CD86 in NLCs (Figure 2C and E, respectively), which perhaps could be suggestive of a general reduction in macrophage activation. Importantly, macrophages expressing high levels of CD163 and CD86 have been observed in diffuse large B cell lymphoma [58], suggesting that the NLC phenotype may be seen in other B-cell malignancies and that AHCC may be effective there as well.

Previously, it has been shown that AHCC could activate MAPK signaling as well as NF-κB [22]. In the THP-1 cell line, the major MAPK activated by AHCC was p38, whereas the MEK pathway was slightly induced [22]. Interestingly, we have previously found that MEK signaling activity, as well as p38 inhibition, induces NLC differentiation [59]. Both MAPKs have been reported to have opposing roles [60,61,62]. Thus, activation of p38 by AHCC may help explain the reduced number of NLCs obtained in culture.

The effects of AHCC against CLL were also tested in vivo. Using the well-established Eµ-TCL1 adoptive transfer model [63], we found that treatment successfully diminishes leukemic burden and increases survival. This effect may be explained in part by the effects on NLCs and direct effects on CLL cells that we observed in vitro. In addition, as previously mentioned, AHCC regulates other important cells involved in the immune response such as NK and T cells, which may have antitumoral effects as well. Regarding our findings in the AML model, the abovementioned activation of myeloid-lineage cells [22,33,34] may suggest that AHCC had a direct effect on the tumor cells. This will require further elucidation.

Of particular importance, AHCC enhanced antitumor antibody treatment in vivo, suggesting that it may be most effective in combination with immune-oriented therapies. AHCC impacts tumor-supportive NLCs, activates immune cells, and directly antagonizes CLL-cell survival. All three elements likely contribute toward the enhancement of antibody effect by AHCC, although further studies will be needed to identify their relative contributions. It is unknown whether AHCC would increase the effectiveness of other therapies. For example, BTK inhibitors have gained prominence as treatments for CLL [64]. Here, AHCC may be of benefit via at least two mechanisms. Firstly, it shows effects against CLL cells, which could sensitize the tumor cells to BTK inhibitors. Secondly, we found that AHCC antagonizes NLC development, and it has been shown that NLCs help protect CLL cells from agents such as ibrutinib [12].

## 5. Conclusions

In conclusion, AHCC has shown efficacy against CLL in vitro and in vivo. This was seen when tested as a single agent and when combined with antitumor antibody. Collectively, these results help support the clinical testing of AHCC as a candidate agent for the treatment of CLL.

## Data Availability

Raw cytometry data are available upon request. The data are publicly available at http://flowrepository.org/id/FR-FCM-Z74Y.

## Supplementary Materials

The following supporting information can be downloaded at: https://www.mdpi.com/article/10.3390/nu15245138/s1.

## Abbreviations

α-CD20, anti-CD20 monoclonal antibodies; AHCC, Active Hexose-Correlated Compound; BCR, B-cell receptor; BSA, Bovine Serum Albumin; BTK, Bruton Tyrosine Kinase; IL-1β, Interleukin 1β; IL-2, Interleukin 2; IL-6, Interleukin 6; IRB, Institutional Review Board; NK, Natural Killer; NKT, Natural Killer T; NLC, Nurse-like cell; PBS, phosphate-buffered saline; PI3K, phosphoinositol-3 kinase; PRR, Pattern Recognition Receptor; RPMI, Roswell Park Memorial Institute; SDF-1, Stromal Cell-Derived Factor 1; STAT, Signal Transducer and Activator of Transcription; Th1, T helper 1; TLRs, Toll-like receptors; TNF-α, Tumor Necrosis Factor α.
