# Supplementary material for: Active Hexose-Correlated Compound Shows Direct and Indirect Effects against Chronic Lymphocytic Leukemia
Source: Nutrients. 2023 Dec 18;15(24):5138. doi: 10.3390/nu15245138 (PMC10747249; doi:10.3390/nu15245138)
Supplement: Supplementary file 1 [file nutrients-15-05138-s001.zip › nutrients-2718304-supplementary.pdf]

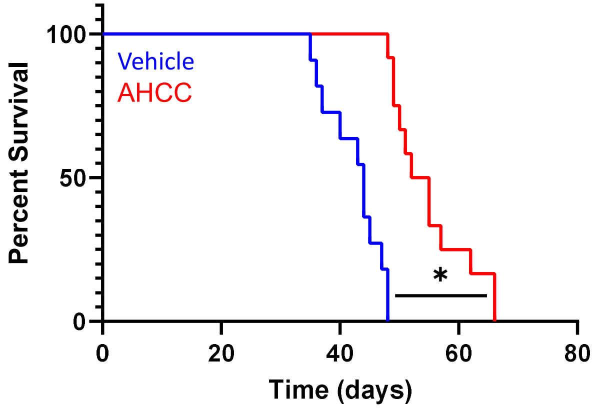

Figure S1. AHCC increases survival in an immune-competent mouse model of AML. 0.75x10^6 $Mll^{PTD/WT}Flt3^{ITD/WT}$ splenocytes from diseased mice (Lys5.2) were injected intravenously into Lys5.1 C57Bl/6J mice. After one week, mice were gavaged 3 times per week with PBS (Vehicle, n=11) or with 600 mg/kg AHCC (n=12). Survival was counted as time until meeting early-removal criteria. * $p \leq 0.0001$.

Patient ID	Age	WBC ($10^3/\mu L$)	Previous Treatment	IGVH mutation status	Cytogenetics
Patient 1	63	24.32	none	Mutated (4.6%)	del13q
Patient 2	78	34.75	none	Mutated (7.9%)	del13q
Patient 3	59	12.32	none	Unmutated (1.4%)	del13q
Patient 4	80	6.24	none	Mutated (6.1%)	trisomy 12
Patient 5	68	25.69	none	Unknown	del13q
Patient 6	76	16.55	none	Unknown	Unknown
Patient 7	51	15.74	none	Mutated (4.8%)	del13q(41.7%)/ nul13q(7.3%)
Patient 8	61	24.40	none	Mutated (9.7%)	del13q
Patient 9	76	12.36	none	Mutated (6.8%)	del13q(73.6%)/ nul13q(11.8%)
Patient 10	73	11.45	none*	Unknown	nul13q

Table S1. Characteristics of CLL patients who donated samples for this study. 5 patients were male and 5 female, and average age was 68.5 ± 9.6 years. * Patient was treated with acalabrutinib and steroids during hospitalization in July 2020 for COVID-19. Patient is considered treatment-naïve for CLL.
